# Clot signature in patients with large vessel occlusion stroke and concomitant active cancer

**DOI:** 10.1111/ene.70037

**Published:** 2025-01-06

**Authors:** Malin Woock, Rosanna Rossi, Duaa Jabrah, Andrew Douglas, Petra Redfors, Annika Nordanstig, Turgut Tatlisumak, Erik Ceder, Dennis Dunker, Jeanette Carlqvist, István Szikora, Georgios Tsivgoulis, Klearchos Psychogios, Georgios Magoufis, Alexandros Rentzos, Karen M. Doyle, Katarina Jood

**Affiliations:** ^1^ Department of Neurology Sahlgrenska University Hospital Gothenburg Sweden; ^2^ Department of Clinical Neuroscience, Institute of Neuroscience and Physiology Sahlgrenska Academy at University of Gothenburg Gothenburg Sweden; ^3^ Department of Physiology and Galway Neuroscience Centre, School of Medicine University of Galway Galway Ireland; ^4^ CÚRAM–SFI Research Centre in Medical Devices University of Galway Galway Ireland; ^5^ Institute of Biotechnology and Biomedicine IBB, Autonomous University of Barcelona Barcelona Spain; ^6^ Department of Radiology, Section of Diagnostic and Interventional Neuroradiology Sahlgrenska University Hospital Gothenburg Sweden; ^7^ Department of Radiology, Institute of Clinical Sciences Sahlgrenska Academy at the University of Gothenburg Gothenburg Sweden; ^8^ Department of Neurointerventions National Institute of Clinical Neurosciences Budapest Hungary; ^9^ Second Department of Neurology National & Kapodistrian University of Athens, “Attikon” University Hospital Athens Greece; ^10^ Stroke Unit Metropolitan Hospital Piraeus Greece

**Keywords:** acute ischemic stroke & cancer, clot composition, collagen, NETS, vWF

## Abstract

**Background and Purpose:**

Patients with active cancer face an increased risk of ischemic stroke. Also, stroke may be an initial indicator of cancer. In patients with large vessel occlusion (LVO) stroke treated with thrombectomy, analysis of the clot composition may contribute new insights into the pathological connections between these two conditions.

**Methods:**

We compared the content of 64 consecutively retrieved clots from LVO stroke patients with concomitant active cancer and 64 clots from matched‐control LVO stroke patients without a history of cancer. Clots were analyzed with respect to histological composition by Martius Scarlet Blue, von Willebrand factor (vWF), citrullinated histone H3 (H3Cit, a biomarker of NETS), CD42b, and CD3 expression by immunohistochemistry. Orbit Image Analysis was used for quantification. Differences between groups were tested using the Mann–Whitney *U*‐test and Chi‐square Test.

**Results:**

Clots from patients with concomitant cancer had a significantly higher content of vWF (median 26 [IQR13‐38]% vs. 10 [4–18]%, *p* < 0.0001) and H3Cit (median 0.11 [IQR0.02–0.46]% vs. 0.05 [0.00–0.28]% *p* = 0.027) than controls. The presence of collagen >1% within the retrieved clots was highly indicative of cancer, occurring in 16/64 with active cancer and in 3/64 controls, *p* = 0.002. After correction for multiple comparisons, the statistical significance for H3Cit was lost. Red and white blood cells, platelets, fibrin, and expression of CD3 and CD42b did not differ between the groups.

**Conclusions:**

Clots from LVO patients with concomitant active cancer possess distinct characteristics, indicating an influence of cancer on the innate immune system, fibroblasts, and the vascular endothelium in the formation of LVO clots.

## INTRODUCTION

Patients with active cancer have substantially increased short‐term risk of ischemic stroke, stroke recurrence, and poor long‐term functional outcomes after stroke [1, 2]. For affected patients, this causes suffering and considerable shortening of their lifetime expectancy. Over the last decade, the proportion of patients with ischemic stroke and concomitant cancer has increased and almost 5% of hospitalized patients with ischemic stroke have active cancer [3, 4]. Although patients with cancer often share the same risk factors as stroke patients, there are specific mechanisms connected to cancer that may cause stroke. Coagulation disturbances have been suggested to be the main mechanism [5], making it plausible that blood clots causing ischemic stroke in patients with concomitant active cancer have a different composition compared to patients without cancer.

The introduction of mechanical thrombectomy for patients with large vessel occlusion (LVO) stroke has substantially improved the clinical outcome for patients with severe ischemic stroke [6, 7]. Besides, clot removal offers the possibility for further histopathological and biochemical analyses giving us vital information about clot structure and composition [8, 9, 10, 11, 12] as well as a unique opportunity to discover novel biomarkers for stroke etiology and management [13, 14]. Since stroke can be the initial manifestation of cancer [15], information about the histopathological and biochemical signatures of clots in ischemic stroke patients with active cancer could potentially improve diagnostics and help select cases in whom cancer screening should be performed. Knowledge of clot histopathological and biochemical signatures may also help to bridge the knowledge gap with respect to the optimal choice for secondary preventive therapy in ischemic stroke patients with concomitant active cancer. This is particularly important given that the underlying stroke mechanism remains unidentified in up to 50% of active cancer patients experiencing an ischemic stroke, despite exhaustive clinical work‐up [16]. Prior studies investigating histological components in clots retrieved from LVO stroke patients indicate different clot profiles for different stroke etiologies. However, until now the results have been conflicting, highlighting the fact that further larger studies are warranted before a definite clot signature can be confirmed [11, 17]. Studies of clot composition in stroke patients with concomitant active cancer are particularly scarce. Higher platelet or fibrin/platelet and lower red blood cell (RBC) fractions were recently reported from two studies [18, 19], indicating that platelet‐related mechanisms may play an important role in cancer‐related LVO stroke. However, these studies were limited by the small number of patients with active cancer (*n* = 19 and 16, respectively).

Here, we build on these results and hypothesize that clots in LVO stroke patients with concomitant cancer differ in composition compared to those in LVO stroke patients without cancer, reflecting a variance in the underlying mechanism. To investigate this, we performed a case–control study, in which we comprehensively assessed the clot composition in a cohort of consecutive patients with LVO stroke and concomitant active cancer (64 patients) compared to matched‐control LVO stroke patients without a history of cancer (64 patients).

It is well known that cancer development and progression are driven by complex interaction between tumor cells and non‐malignant host cells. Above all, the interaction between platelets and neutrophils by NETosis‐web‐like structures of externalized chromatin and proteases has previously been described to promote cancer‐associated thrombosis and inflammation [20]. In addition, cancer‐associated fibroblasts and distinct populations of T‐cells have been recognized as key players for the cancer microenvironment composition and tumorigenesis [21]. We therefore specifically studied the histological composition and markers of endothelial damage, platelets, T‐cells, and neutrophil extracellular traps (NETs) by immunohistochemistry (IHC).

## METHODS

### Patient selection and clinical data

We performed a case–control study based on a multi‐center registry with prospectively collected clots retrieved from LVO stroke patients within the RESTORE registry [22].

The RESTORE registry is a record of thrombotic material extracted via mechanical thrombectomy from patients with acute LVO stroke. Our study included patients from three of the participating European stroke centers. All patients were aged ≥18 years and had acute ischemic LVO stroke from the anterior or posterior circulation, treated by mechanical thrombectomy between the period March 2018 and October 2021 [22]. The study was performed in accordance with the Strobe guidelines [23] and ethical standards of the Declaration of Helsinki and its amendments [24]. The University of Galway research ethics committee (16‐SEPT‐08) and the regional or hospital ethics committees (Regional Ethical Board in Gothenburg; National Institute of Clinical Neuroscience, Budapest; Metropolitan Hospital, Athens) approved the study. Written informed consent or a waiver of consent was obtained from the patients for participation in this study. All medical records were reviewed up to 6 months after stroke to identify patients who developed cancer within 6 months after stroke index. For patients with a cancer diagnosis, we collected data on the date of diagnosis, location, type of cancer, presence of metastasis, and details on cancer treatment from medical records or by a telephone interview at follow‐up. Clots from all patients fulfilling the criteria for active cancer (defined as either a new cancer diagnosis, metastasis of known cancer, recurrent cancer, or receiving cancer treatment, all within 6 months before or after stroke onset) were included. However, clots from cancer patients with only hormonal cancer treatment, patients with meningioma, non‐invasive skin cancer, or myelodysplastic syndrome were excluded. For each patient fulfilling the criteria for active cancer, one investigator, blinded to clinical data, randomly selected one sex‐and age‐matched control among LVO patients in the RESTORE registry. To ensure cancer‐free status among controls, only patients treated in Gothenburg, Region Västra Götaland, Sweden were eligible as controls. For these patients, we had access to electronic medical records from all hospitals in the Region Västra Götaland. According to local routine, patients with suspected malignancy are referred to the hospital for diagnostic work‐up, cancer diagnosis, and treatment. We checked the cancer‐free status of all selected controls. If a diagnosis of inactive cancer was identified, the control was excluded, and we randomly selected a new one.

For all patients, information on demographics, pre‐stroke modified Rankin scale (mRS), vascular risk factors, comorbidity including cancer diagnosis, admission National Institutes of Health Stroke Scale (NIHSS) score, medication and blood chemistry at baseline, administration of intravenous thrombolysis (IVT), and details about the endovascular procedure were collected from medical records. Etiology was classified according to the TOAST criteria [25]. We used the specific criteria for cancer‐related pro‐thrombotic state stroke, previously defined by Costamagna et al. [2]. The extent of reperfusion was investigated by anterioposterior/lateral digital subtraction angiography and individually graded by two radiologists at every treatment center according to the final expanded Treatment in Cerebral Ischemia (eTICI) score [26].

### Clot processing and histological characterization

Endovascular treatment was performed according to the local routine at every treatment center. The clots retrieved during thrombectomy were collected per‐pass, meaning if multiple passes were required during treatment, clot material was collected separately at every pass.

Clot material was immediately placed in 10% formalin pots and sent to the core laboratory in Galway for analysis. On‐site, gross photographs were taken of each clot using a Canon EOS 1300D camera by pathologists blinded to the clinical characteristics of the patients, including concomitant cancer. ImageJ software https://imagej.net/ij/index.html was used to analyze the clot area for each extracted clot fragment individually, obtaining the Extracted Clot Area (ECA), as previously described [27, 28]. Briefly, the procedure works as follows: the scale was set in the software for the clot image; the polygon tool was used to draw around the fragment of interest obtaining in this way the area of the fragment. The total ECA for each case was calculated as the sum of the areas of all the fragments.

**FIGURE 1:**
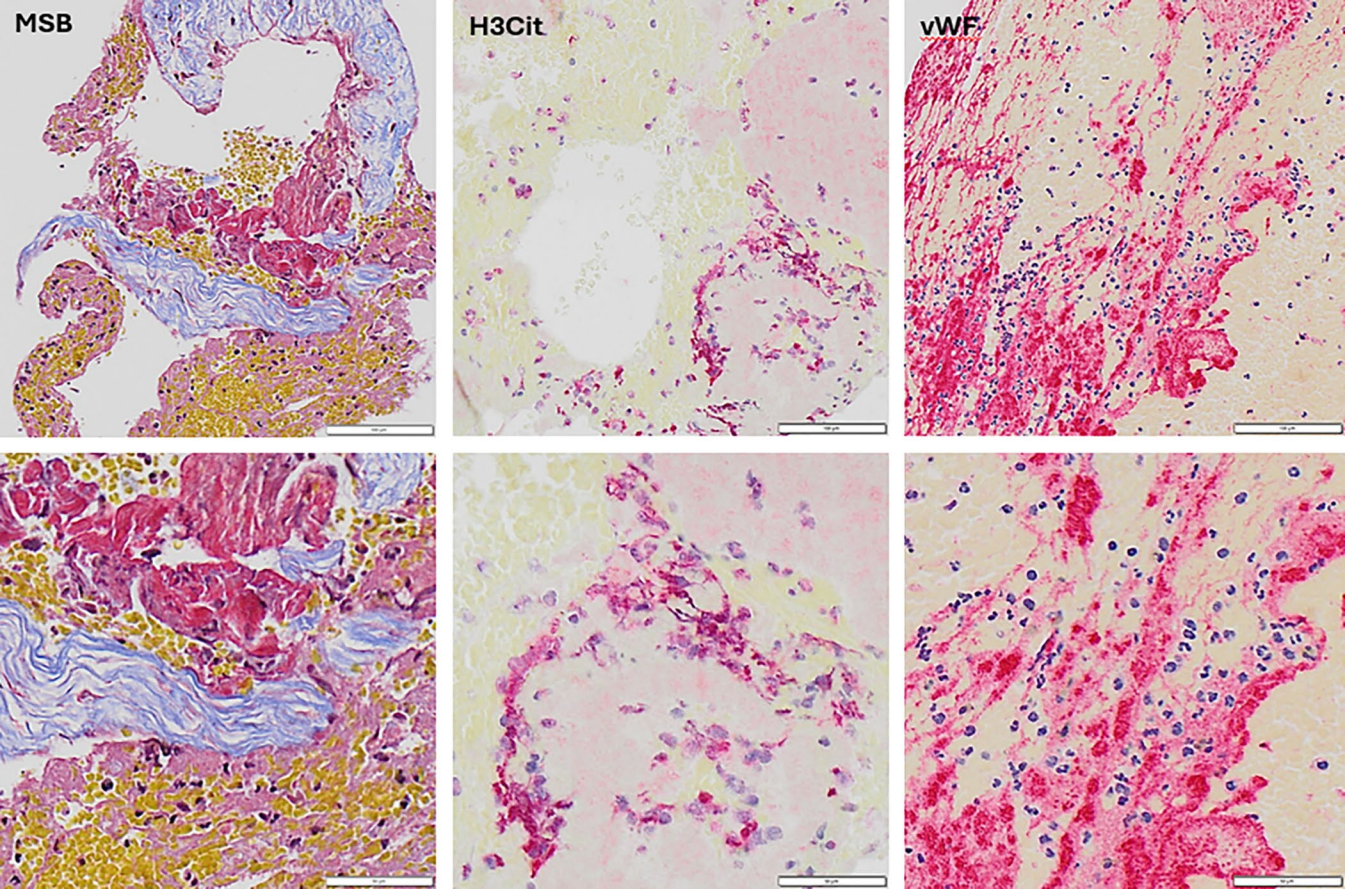
Shows representative images following Martius Scarlet Blue (MSB), citrullinated histone H3 (H3Cit), or von Willebrand factor (vWF) staining. Images were captured at 20× magnification. MSB‐stained images show red blood cells (yellow), fibrin (red), platelets (grey‐pink), white blood cells (dark blue), and collagen (light blue). Immuno‐stained images show H3Cit or vWF stained red, with hematoxylin (dark blue) counterstaining of nucleated cells.

The clots were thereafter paraffin‐embedded, cut with a microtome in 3 μm sections, and stained with Martius Scarlett Blue (MSB) for histological characterization. This staining identifies the main histological components of clot tissue as previously described [29]: Red blood cells (RBCs) are stained in yellow; nucleated cells (NUCs), including white blood cells, are stained in blue; fibrin (FIB) is stained in red; platelets are stained in gray/pink (PTL) and collagen is stained in light blue (Figure 1).

### Immunohistochemistry staining

Immunohistochemistry staining was performed to evaluate the expression of specific markers: von Willebrand factor (vWF), marker of endothelial damage and thrombotic risk [30]; the platelet marker CD42b; citrullinated histone H3 (H3Cit), a marker of NETs and CD3, a marker of T cells.

Immunohistochemistry staining of 3 μm sections was performed on a Leica Bond‐III autostainer. The specific conditions used for each antibody are reported in supplement as Table S1. Primary antibody dilutions were all performed using BOND Primary Antibody Diluent (#AR9352) and incubation time was 15 min, followed by 30 min of incubation with an anti‐rabbit secondary antibody. Visualization of the staining was performed using the Bond Polymer Refine Detection Kit (DS9800) and the Bond Polymer Refine Red Detection Kit (DS9390). Counterstaining of tissue using hematoxylin was performed for 5 min. Sections were then washed with a washing solution (Leica Biosystems #AR9590) and rinsed in distilled water. Sections were then dehydrated in alcohol, cleared in xylene, and mounted with DPX. Negative controls were performed by omission of the primary antibody step.

### Slide scanning and quantification

Stained slides were scanned on an Olympus vs120 slide scanner at 20× magnification and digital whole slide scan images were generated. To quantify the histological and immunohistochemical clot components we used the quantification tool Orbit image analysis (www.orbit.bio) as previously described [22].

Briefly, exclusion and inclusion models were created to distinguish regions to be excluded (e.g., background and artifact) and regions containing the component (s) of interest, enabling their quantitative assessment within each clot.

Clot composition, both histological and IHC analysis was calculated in this way:

$$\text{Example}:\%\mathrm{RBC}\;\text{case}=\left(\%{{\text{RBCP}}_{1}}^{*}{\text{ECAP}}_{1}+\%{{\text{RBCP}}_{2}}^{*}{\text{ECAP}}_{2}+\ldots \%{{\text{RBCP}}_{N}}^{*}{\text{ECAP}}_{N}\right)/\text{ECAtot}$$

Where P_N_ is the pass number and ECAtot is the ECA of the whole case. A similar calculation was performed for the other components.

### Statistical analysis

IBM SPSS Statistics 25 was used to assess all statistical correlations. Clinical demographic data were reported as mean ± standard deviation if data followed Gaussian distribution and otherwise as median and quartiles or number (%) of cases. Histological and IHC components were reported as mean ± standard deviation and median [IQ1–IQ3] (%). The Shapiro–Wilk test indicated that quantitative values did not follow a standard normal distribution. We therefore used the non‐parametric Chi‐square Test and Mann–Whitney *U*‐test to assess differences between the groups. A level of statistical significance for all analyses was set at *p* < 0.05 (two‐sided). For the correction of multiple testing, we used the Bonferroni and Benjamini‐Hochberg procedures.

## RESULTS

### Baseline characteristics of LVO patients

We included 64 patients with LVO stroke fulfilling the criteria for concomitant active cancer and 64 sex‐and age‐matched LVO stroke patients without a history of cancer. The clinical and procedural characteristics of patients with and without cancer are presented in Table 1. The mean age in patients with active cancer was 72 ± 10, and in patients without cancer 71 ± 11, 50% were men. Patients without concomitant cancer had a nominally higher proportion of previous stroke, a nominally higher frequency of typical ischemic stroke risk factors, a nominally higher admission NIHSS scores, and a nominally higher proportion of T‐occlusions. Among eight patients with concomitant active cancer classified as having an “other determined etiology of stroke” according to TOAST criteria, the etiology was marantic endocarditis in one, and cancer‐related pro‐thrombotic state stroke in five. Another four patients fulfilling the criteria for a cancer‐related pro‐thrombotic state presented a concomitant cause for stroke and were therefore included in the group of “undetermined etiology”.

**TABLE 1:** Baseline and procedural characteristics of LVO stroke patients with and without active cancer.

	Active cancer *N* = 64	No cancer *N* = 64
Age in years—mean ± SD	72.1 ± 10.3	71.0 ± 10.8
Male sex—*n*/*N* (%)	32/64 (50)	32/64 (50)
Admission NIHSS—median [Q1‐Q3]	15 [12–20]	18 [12–22]^a^
Onset of stroke—*n*/*N* (%)		
Known	29/60 (48.3)	37/64 (57.8)
Estimated	16/60 (26.7)	11/64 (17.2)
Wake up stroke	15/60 (25.0)	16/64 (25.0)
Medical history—*n*/*N* (%)		
Previous stroke	7/64 (10.9)	12/64 (18.8)
Hypertension	34/64 (53.1)	40/64 (62.5)
Diabetes mellitus	10/64 (15.6)	15/64 (23.4)
Coronary heart disease	11/64 (17.2)	17/64 (26.6)
Hypercholesterolemia	15/64 (23.4)	13/64 (20.3)
Atrial fibrillation	21/64 (32.8)	26/64 (40.6)
Smoking	6/47 (12.8)	7/50 (14.0)
Laboratory markers—median [Q1–Q3]		
Hemoglobin, g/L	121.0 [108.0–137.5]^b^	130.0 [118.0–143.0]^c^
Hematocrit, L/L	0.38 [0.34–0.42]^d^	0.41 [0.39–0.44]^e^
White blood cells, ×10^9^/L	10.3 [7.6–12.6]^a^	10.5 [8.7–12.6]^f^
Platelets, ×10^9^/L	224.0 [174.3–318.3]^a^	231.5 [194.3–285.0]
aPTT, seconds	25.0 [24.0–28.2]^g^	25.0 [23.8–27.3]^a^
Prohrombin time, INR	1.1 [1.0–1.2]^g^	1.1 [1.0–1.1]^a^
C‐reactive protein, mg/L	15.0 [4.5–40.5]^b^	7.0 [2.0–16.0]
Glucose, mmol/L	6.8 [5.7–7.7]^c^	7.0 [6.0–8.9]^e^
Medication—*n*/*N* (%)		
Statins	20/63 (31.7)	24/64 (37.5)
Anticoagulation therapy	24/64 (37.5)	17/64 (26.6)
Antiplatelet therapy	14/64 (21.9)	17/64 (26.6)
Ischemic stroke subtype (TOAST)—*n*/*N* (%)		
Large‐artery atherosclerosis	11/64 (17.2)	12/64 (18.8)
Cardioembolism	21/64 (32.8)	32/64 (50.0)
Other determined etiology	8/64 (12.5)^h^	2/64 (3.1)
Undetermined etiology	24/64 (37.5)^i^	18/64 (28.1)
Thrombolysis—*n*/*N* (%)	13/64 (20.3)	17/64 (26.6)
Interventional parameters—*n*/*N* (%)		
Single occlusion	59/64 (92.2)	53/64 (82.8)
Multiple occlusion	5/64 (7.8)	11/64 (17.2)
Single occlusion site, *n*/*N* (%)		
T–occlusion	3/64 (4.7)	7/64 (10.9)
ICA	6/64 (9.4)	5/64 (7.8)
MCA (M1, M2 and M3)	44/64 (68.8)	36/64 (56.2)
PCA (P1 and P2)	1/64 (1.6)	1/64 (1.6)
Basilar	2/64 (3.1)	2/64 (3.1)
Tandem occlusion	3/64 (4.7)	2/64 (3.1)
No. of passes, median [Q1–Q3]	2 [1–3]	2 [1–4]
Aspiration as first method—*n*/*N* (%)	52/57 (91.2)	64/64 (100)
Final eTICI score, *n*/*N* (%)		
eTICI 0‐2a	4/64 (6.3)	9/64 (14.1)
eTICI 2b	17/64 (26.6)	24/64 (37.5)
eTICI 2c	20/64 (31.3)	10/64 (15.6)
eTICI 3	23/64 (35.9)	21/64 (32.8)
Time from vessel puncture to recanalization, median [Q1–Q3] h:mm	0:27 [0:15–00:59]^g^	0:46 [0:25–1:21]^a^
Time from stroke onset to recanalization, median [Q1–Q3] h:mm	3:50 [2:49–4:21]^j^	4:02 [3:00–5:09]^j^

Abbreviations: ICA, internal carotid artery; eTICI, expanded Treatment in Cerebral Ischemia; MCA, middle cerebral artery; NIHSS, National Institutes of Health Stroke Scale; PCA, posterior cerebral artery; SD, Standard deviation.

^a^ Value missing in 2.

^b^ Value missing in 3.

^c^ Value missing in 9.

^d^ Value missing in 5.

^e^ Value missing in 13.

^f^ Value missing in 1.

^g^ Value missing in 4.

^h^ One patient with marantic endocarditis and 5 with cancer‐related hypercoagulability according to criteria(2).

^i^ Four patients with cancer‐related hypercoagulability according to criteria and at least one concomitant etiology.

^j^ Represent only cases with known stroke onset time.

Procedure time was nominally longer among patients without cancer compared to patients with active cancer. However, the recanalization outcome after the procedure appeared similar for the two groups in terms of final eTICI score, and similar total number of passes.

### Characteristics of active cancer

Colorectal cancer and genitourinary cancer were the most common cancer types, followed by lung and pancreas cancer (Table 2). The majority presented with advanced‐stage disease; 63% had known metastasis at stroke onset. However, almost 19% had occult cancer detected at stroke hospitalization or follow‐up. One third of patients had active cancer treatment at the time of their stroke.

**TABLE 2:** Characteristics of cancer in patients with active cancer.

	*n*/*N* %
Cancer type	
Colorectal	17/64 (26.6)
Genitourinary	13/64 (20.3)
Lung/larynx	12/64 (18.8)
Pancreas	7/64 (10.9)
Breast	3/64 (4.7)
Biliary	2/64 (3.1)
Gastric/esophageal	2/64 (3.1)
Hematologic	2/64 (3.1)
Skin	2/64 (3.1)
Multiple^a^	2/64 (3.1)
Other^b^	2/64 (3.1)
Presence of metastasis at stroke onset	34/54 (63.0)
Occult cancer^c^	12/64 (18.8)
Ongoing treatment for cancer at stroke onset^d^	21/61 (34.4)
Chemotherapy	8/61 (13.1)
Radiation	2/61 (3.3)
Surgery	4/61 (6.6)
Targeted cancer therapy^e^	7/61 (11.5)

^a^ Including one patient with breast and lung cancer and one patient with KLL and corpus uteri cancer.

^b^ Including one patient with myxoma and one patient with an unclear primary tumor.

^c^ Patients with cancer diagnosed after stroke onset.

^d^ Ongoing chemotherapy or targeted cancer therapy, prior neck radiation before stroke onset, or surgery within 2 weeks.

^e^ Protein kinase inhibitors and immune therapy.

### Histological and IHC analysis: clots from patients with active cancer have more collagen, vWF, and H3Cit than control patients

We analyzed a total of 195 clots, 95 from patients with active cancer and 100 clots from control patients. Martius Scarlett Blue showed that clots from the active cancer group have a higher content of collagen compared to controls (Table 3). Collagen was rarely detected in clots from patients without cancer; only 3 out of 64 (4.7%) had a collagen content of ≥1%, while in patients with active cancer, 16 out of 64 patients (25%) had clots with a collagen content equal or above this value (*p* = 0.002, Fisher's exact test), corresponding to a positive predictive value of 84%. Sub‐analysis showed that among active cancer patients with elevated values of collagen, 86% had advanced disease with the presence of metastases compared to only 14% with elevated values of collagen but no signs of metastasis. This difference was not statistically significant (*p* = 0.056, Fisher's exact test).

**TABLE 3:** Histological, Immunohistochemical composition, and Extracted Clot Area of clots from cancer patients versus control patients.

Component	Cancer cases (*N* = 64)	Control cases (*N* = 64)	*p*‐value
	MSB		
	Median[Q1–Q3]	Median[Q1–Q3]	
RBCs	37 [13–62]%	42 [27–60]%	0.326
NUCs	3 [1–5]%	2 [2–4]%	0.371
FIB+PTL	56 [29–80]%	55 [38–70]%	0.915
Collagen	0.00 [0.00–0.50]%	0.00 [0.00–0.00]%	0.001

Component	IHC		*p*‐value
	Median[Q1–Q3]	Median[Q1–Q3]	
vWF	26 [13–38]%	10 [4–18]	<0.0001
H3Cit	0.11 [0.02–0.46]%	0.05 [0.00–0.28]%	0.027
CD42b	18 [6–38]%	14 [7–24]%	0.273
CD3	0.12 [0.05–0.27]%	0.13 [0.08–0.25]%	0.590

	ECA (mm^2^)		*p*‐value
	Median[Q1–Q3]	Median[Q1–Q3]	
	40[22–78]	37[17–86]	0.488

*Note*: Differences between groups were tested using the Mann–Whitney *U*‐test. *p* < 0.05, *p* < 0.001.

Abbreviations: CD3, Cluster of differentiation 3; CD42b, Cluster of differentiation 42b; ECA, Extracted Clot Area; FIB, Fibrin; H3Cit, citrullinated histone H3; IHC, immunohistochemistry; MSB, Martius Scarlett Blue; NUCs, nucleated cells; PTL, platelet and other components; RBCs, red blood cells; vWF, von Willebrand Factor.

No significant differences were found for the percentage of RBCs, NUCs, and FIB+PTL in clots between the two groups.

Immunohistochemistry analysis showed that clots from cancer cases expressed a significantly higher percentage of vWF (median[Q1–Q3] 26 [13–38]% vs. 10 [4–18]%, *p* < 0.0001) and H3Cit (median[Q1‐Q3] 0.11 [0.02–0.46]% vs. 0.05 [0.00–0.28]% *p* = 0.027) than controls, while no difference was found in terms of CD42b and CD3 expression between the two groups (Table 3). After correction for multiple comparisons, collagen and vWF expression differences remained statistically significant, while the p‐value for H3Cit did not reach the adjusted level of significance (Tables S2 and S3). No difference was observed in terms of the size of extracted clots, expressed as ECA, between the two groups (Table 3).

## DISCUSSION

In this study of retrieved clots from patients with LVO stroke, we found that clots from patients with concomitant active cancer displayed a distinct pattern compared to patients without cancer, with higher expression of vWF and H3Cit. Moreover, the presence of collagen within the extracted clot was highly indicative of underlying active cancer. In contrast, the content of white blood cells, platelets, and fibrin and the expression of CD3 or CD42 did not differ significantly between the two groups. The content of RBCs was lower in the clots from patients with active cancer, but the difference was not statistically significant.

Von Willebrand factor, an acute phase response protein and vital in the hemostatic process, is under normal circumstances carefully regulated in the body since too little or malfunctional protein will lead to different forms of the well‐known bleeding disorder von Willebrand disease [31]. On the contrary, high levels or too little degradation of the glycoprotein will inevitably lead to undesired thrombogenicity with an increased risk of stroke alone or as part of the life‐threatening thrombotic microangiopathy, thrombotic thrombocytopenic purpura [32, 33]. Interestingly, IHC in our study showed a significant increase of vWF expression in LVO clots from patients with active cancer compared to controls. Previous studies in mouse and human melanoma showed that vWF plays a key role in promoting cancer‐associated platelet aggregation [34], which is in line with results from studies showing increased platelet aggregation in patients with various types of cancer [35, 36]. Furthermore, there is evidence that high vWF levels in plasma and tumor tissue of patients with cancer can predict cancer‐associated venous thromboembolism and mortality [37, 38]. By providing a possibility for cancer cells to interact with platelets and with the endothelium, vWF is thought to contribute to cancer spread and metastasis [32]. Even though preclinical studies have shown conflicting results [34, 39], clinical studies have consistently demonstrated increased levels of vWF in patients with different malignancies with higher levels for patients with distant metastasis [40, 41]. Thus, our results showing high content of vWF in clots retrieved from stroke patients with concomitant cancer, lend new and further support to the previous literature suggesting a key role of vWF in the formation of clots in patients with active cancer.

The higher content of H3Cit in LVO clots from patients with active cancer did not reach statistical significance after correction for multiple comparisons. However, the involvement of H3Cit in cancer‐associated thromboembolic events is biologically plausible [20]. Moreover, in line with our observation, Thalin et al. [42] reported higher levels of H3Cit in both plasma and clot content from multiple organs in autopsy specimens of patients with acute ischemic stroke and active cancer, supporting the hypothesis of a hypercoagulable state driven by NETosis. In the cancer microenvironment, neutrophils play a crucial role as they are stimulated to promote inflammation through the production of cytokines and reactive oxygen species and to release increased amounts of NETs serving as a scaffold for thrombocyte adhesion and aggregation promoting thrombus formation [43]. In our study, patients with active cancer also had nominally higher levels of CRP compared to controls, which is consistent with previous studies on stroke patients with active cancer [44, 45]. Interestingly, the C‐reactive protein has also been recognized as a biomarker of inflammation involved in vascular disorders, reportedly associated with NETosis induction [46]. Therefore, taken together, we believe that the finding of higher expression of H3Cit in LVO clots from stroke patients with concomitant cancer, although not statistically significant after correction for multiple testing, lends support to the hypothesis that NETosis is involved in the formation of clots in patients with cancer suffering a LVO stroke.

The tumor microenvironment has shown to have a remarkable impact also on the extracellular matrix (consisting mainly of different components of collagen) and on the fibroblasts residing in the interstitial matrix [21]. Cancer cells can interrupt the homeostasis of the basal membrane by secreting high amounts of metalloproteinases [21], causing degradation, remodeling, and dysfunction of the basal membrane. Emerging evidence also shows that transformed fibroblasts, so‐called cancer‐associated fibroblasts, and the collagens they secrete, play a key role in tumorigenesis, cancer progression, and metastasis [47]. Our results indicate that these processes may also be of importance for clot formation, as collagen content in clots were highly eloquent for concomitant active cancer.

Two small previous studies investigating clot content in patients with stroke and concomitant active cancer reported lower content of RBCs and higher content of platelets and/or fibrin [18, 19]. In line with these findings, the content of RBCs was somewhat lower, and CD42b somewhat higher in patients with active cancer, although the difference was not as large as in previous studies and not statistically significant. There are possible explanations for the somewhat different findings on clot composition between our study and the previous ones. The association between cancer and stroke is complex and may be mediated by diverse mechanisms [1, 4], probably depending on cancer type, stage, and cancer treatment. More than half of the cancer patients in our cohort received anticancer therapy, and we cannot exclude a possible synergic effect of anticancer treatment in increasing stroke risk. In fact, it has been demonstrated that some chemotherapy agents, such as methotrexate, 5‐fluorouracil, cisplatin, and L‐asparaginase might lead to stroke via endothelial toxicity and abnormalities in coagulation and hemostasis factors, although, the risk of a chemotherapy‐induced stroke is regarded as rather low [1, 48]. Similarly, radiotherapy has also been implicated in increasing stroke risk both in the short and long term for cancer patients [49].

Moreover, cancer may be an innocent bystander in some, as cancer and stroke partly share risk factors. Lastly, anticoagulant and antiplatelet treatment already before stroke onset may also influence clot composition. Thus, given the limited study samples in all studies, differences in case mix may contribute to diverging results. Future larger studies powered for separate analyses of clots from different cancer types and treatments are therefore warranted.

We found no difference in retrieved clot area among patients with active cancer compared to cancer‐free controls and the number of attempts during thrombectomy did not seem to differ between the groups. However, in contrast to previous findings on stroke in patients with active cancer [50], the proportion of patients with multiple occlusions was nominally lower compared to controls, probably contributing to longer procedure times and somewhat lower recanalization rate among LVO patients without cancer. Since only patients with retrieved clot material were included in this study, it is likely that patients with active cancer and indications for a technically challenging thrombectomy procedure had a lower chance of having clot material retrieved, either because of a lower chance to be accepted for thrombectomy or because of technical difficulties to retrieve clot material.

Taken together, our findings indicate that a cancer‐driven effect specifically on the primary hemostasis, mainly involving interaction between platelets, adhesive proteins located in the subendothelial matrix (including collagen and von Willebrand factor), and the innate immune system is involved in the formation of LVO clots in patients with cancer.

### Limitations

Our study has some limitations. First, only patients with concomitant cancer and LVO stroke accepted for thrombectomy were included and, therefore, our results should not be generalized to all patients with cancer‐related stroke or palliative cancer patients. Furthermore, only retrieved clots could be analyzed. However, to the best of our knowledge, this is the study with the largest sample of cancer‐related clots analyzed to date. Despite this, the sample size was limited, restricting statistical power, and not allowing for separate investigation of clots in different types, stages, or treatment of cancer.

Another limitation is that we could not differentiate the types of collagens present in the clots from patients with active concomitant cancer, since we employed MSB staining to identify every component of the histological clots, including collagen. However, this is definitively worthy of further investigation as it may advance our understanding of the underlying mechanisms.

## CONCLUSIONS

Taken together, we found that LVO clots from patients with concomitant active cancer differ in composition compared to non‐cancer‐related LVO clots, possibly deriving from a cancer‐driven effect on the innate immune system, fibroblasts, and the vascular endothelium. However, further studies in larger samples are needed to address the heterogeneity with respect to cancer types, stages, mechanisms, and cancer treatment.

## AUTHOR CONTRIBUTIONS

**Malin Woock:** Writing – original draft; writing – review and editing; conceptualization; validation; data curation. **Rosanna Rossi:** Conceptualization; methodology; validation; writing – original draft; writing – review and editing; formal analysis. **Duaa Jabrah:** Formal analysis; methodology. **Andrew Douglas:** Formal analysis; methodology. **Petra Redfors:** Writing – review and editing. **Annika Nordanstig:** Conceptualization; writing – review and editing; methodology; data curation. **Turgut Tatlisumak:** Conceptualization; investigation; funding acquisition; writing – review and editing. **Erik Ceder:** Data curation; investigation. **Dennis Dunker:** Data curation; investigation. **Jeanette Carlqvist:** Data curation; investigation. **István Szikora:** Investigation; data curation. **Georgios Tsivgoulis:** Investigation; data curation. **Klearchos Psychogios:** Data curation; investigation. **Georgios Magoufis:** Data curation; investigation. **Alexandros Rentzos:** Investigation; conceptualization; data curation; software. **Karen M. Doyle:** Methodology; validation; writing – review and editing; formal analysis; supervision; conceptualization; funding acquisition. **Katarina Jood:** Conceptualization; funding acquisition; writing – review and editing; data curation; supervision.

## FUNDING INFORMATION

This work was supported by academic grants from the Swedish state under the agreement between the Swedish government and the county councils, the ALF agreement (ALFGBG‐965417) and (ALFGBG‐965925), and the Science Foundation Ireland (SFI) and the European Regional Development Fund (Grant Number 13/RC/2073_P2).

## CONFLICT OF INTEREST STATEMENT

TT: has served/serves on scientific advisory boards for Astra Zeneca, Bayer, Boehringer Ingelheim, Bristol Myers Squibb, Inventiva, and Portola Pharm. KJ: has served at scientific advisory board for Janssen. The other authors have no conflict of interest to declare.

## ETHICS STATEMENT

The study was approved by the University of Galway research ethics committee (16‐SEPT‐08) and the regional or hospital ethics committees (Regional Ethical Board in Gothenburg; National Institute of Clinical Neuroscience, Budapest; Metropolitan Hospital, Athens) following the ethical standards of the Declaration of Helsinki.

## Supporting information


**Data S1:** Supporting Information.

**Data S2:** Supporting Information.

## Data Availability

Data may be available upon reasonable request.
