# Supplementary material for: Clot signature in patients with large vessel occlusion stroke and concomitant active cancer
Source: Eur J Neurol. 2025 Jan 6;32(1):e70037. doi: 10.1111/ene.70037 (PMC11702498; doi:10.1111/ene.70037)
Supplement: Supplementary file 1 — Data S1: Supporting Information. [file ENE-32-e70037-s002.docx]

Supplementary material

**CLOT SIGNATURE IN PATIENTS WITH LARGE VESSEL OCCLUSION STROKE AND CONCOMITANT ACTIVE CANCER**

**Authors and affiliations**

Malin Woock^1,2^*, Rosanna Rossi^3,4,5^*, Duaa Jabrah^3^, Andrew Douglas^3,4^, Petra Redfors^1,2^, Annika Nordanstig^1,2^, Turgut Tatlisumak^1,2^, Erik Ceder^6,7^, Dennis Dunker^6,7^, Jeanette Carlqvist^6,7^, István Szikora^8^, Georgios Tsivgoulis^9^, Klearchos Psychogios^10^, Georgios Magoufis^10^, Alexandros Rentzos^6,7^, Karen M. Doyle^3,4^, Katarina Jood^1,2^.

* These authors equally contributed to the manuscript

^1^Department of Neurology, Sahlgrenska University Hospital, Gothenburg, Sweden, ^2^Department of Clinical Neuroscience, Institute of Neuroscience and Physiology, Sahlgrenska Academy at University of Gothenburg, Gothenburg, Sweden,^3^Department of Physiology and Galway Neuroscience Centre, School of Medicine, University of Galway, University Road, Galway, Ireland, ^4^CÚRAM–SFI Research Centre in Medical Devices, University of Galway, Galway, Ireland, ^5^Institute of Biotechnology and Biomedicine, IBB, Autonomous University of Barcelona, Spain ^6^Department of Radiology, Section of diagnostic and interventional neuroradiology, Sahlgrenska University Hospital, Västra Götalandsregionen, Gothenburg, Sweden, ^7^Department of Radiology, Institute of Clinical Sciences, Sahlgrenska Academy at the University of Gothenburg, Gothenburg, Sweden, ^8^National Institute of Clinical Neurosciences, Department of Neurointerventions, Budapest, Hungary, ^9^Second Department of Neurology, National & Kapodistrian University of Athens, “Attikon” University Hospital, Athens, Greece, ^10^Metropolitan Hospital, Stroke Unit, Piraeus, Greece

**Corresponding author:**Malin Woock
Blå stråket 7, 41346 Göteborg, Sweden
malin.woock@vgregion.se

**Table S1. Experimental conditions for IHC staining**

| **Antibody** | **Host** | **Code** | **Brand** | **Dilution** | **Epitope Retrieval (ER)** | **ER time (minutes)** | **Kit** | **Positive CTRL** |
| --- | --- | --- | --- | --- | --- | --- | --- | --- |
| CD42b | rabbit | Ab227669 | Abcam | 1:200 | tris-EDTA^†^ | 20 | Red kit^‡^ | Clot |
| vWf | rabbit | A008202-2 | Dako | 1:200 | NO | No ER | Red kit^‡^ | Clot |
| H3Cit R2+R8+R17 | rabbit | Ab5103 | Abcam | 1:200 | tris-EDTA^†^ | 20 | Red kit^‡^ | Clot with suspected NETS |
| CD3 | rabbit | Ab5690 | Abcam | 1:100 | tris-EDTA^†^ | 20 | DAB^§^ | Tonsil^¶^ |

^†^Leica Biosystems #AR9640; ^‡^BOND Polymer Refine Red Detection kit (Leica Biosystems #DS9390); ^§^BOND Polymer Refine Detection kit (Leica Biosystems #DS9800); ^¶^(BioIVT)

**Table S2. Bonferroni correction**

| \| *Marker* \| \| --- \| | *P-value* | *P-value, adjusted level for significance** |
| --- | --- | --- | --- |
| **H3** | 0,027 | 0,00625 |
| **CD3** | 0,599 | 0,00625 |
| **vWF** | <0.0001 | 0,00625 |
| **CD42b** | 0,273 | 0,00625 |
| **RBC** | 0,326 | 0,00625 |
| **WBC** | 0,371 | 0,00625 |
| **FIB+PTL** | 0,915 | 0,00625 |
| **Collagen** | 0,001 | 0,00625 |

*Bonferroni adjusted level for significance (8 tests)

**Table S3. Benjamini-Hochberg correction**

| \| *Marker* \| \| --- \| | *P-value* | *Rank* | *BH-critical value** |
| --- | --- | --- | --- | --- |
| **H3** | 0,027 | 3 | 0,01875 |
| **CD3** | 0,599 | 7 | 0,04375 |
| **vWF** | <0.0001 | 1 | 0,00625 |
| **CD42b** | 0,273 | 4 | 0,025 |
| **RBC** | 0,326 | 5 | 0,03125 |
| **WBC** | 0,371 | 6 | 0,0375 |
| **FIB+PTL** | 0,915 | 8 | 0,05 |
| **Collagen** | 0,001 | 2 | 0,0125 |

*Benjamini-Hochberg adjusted level for significance (8 tests)
